# Function-preserving microsurgical resection of a massive sporadic cervicothoracic dumbbell neurofibroma: a case report

**DOI:** 10.1093/jscr/rjag426

**Published:** 2026-06-10

**Authors:** Fazeela Bibi, Janmejay Kumar Singh, Khalil El abdi, Ijaz ul Haque, Muhammad Ibrahim, Ahmad Sanan, Gauhar Asghar, Rubbia Sabir, Kanza Ahmed, Sumbal Niaz Afridi, Vohra Maham Hassan, Bilal Aslam, Said Hamid Sadat, Mohammad Mansoor Hassan

**Affiliations:** Jinnah Medical and Dental College, 22-23 Shaheed-e-Millat Rd, Bihar Muslim Society BMCHS Sharafabad, Karachi, 74800, Pakistan; Teerthankar Mahaveer Medical College and Research Center, Moradabad N.H.-9, Delhi Road, Moradabad - 244001, Uttar Pradesh, India; Faculty of Medicine and Pharmacy of Rabat, Mohammed V University, Avenue Mohammed Belarbi El Alaoui, BP 6203, Rabat Institutions, Souissi, Rabat 10100, Morocco; Lady Reading Hospital, Department of Neurology, Peshawar Pakhtunkwa 25000, Pakistan; Bannu Medical College, Bannu, Pakistan; Khyber Medical College, Peshawar, Pakistan; United Medical and Dental College, Township Road PO 28100, Karachi, Pakistan; Allama Iqbal Medical College, Allama Shabbir Ahmed Usmani Road, Lahore, Punjab 54700, Pakistan; United Medical and Dental College, Township Road PO 28100, Karachi, Pakistan; Bannu Medical College, Bannu, Pakistan; Jinnah Medical and Dental College, 22-23 Shaheed-e-Millat Rd, Bihar Muslim Society BMCHS Sharafabad, Karachi, 74800, Pakistan; University of Lahore, 1 km Bhoptian Chowk, Defence Road, Lahore, Punjab 54000, Pakistan; Kabul University of Medical Science Abu Ali Ibn Sina, University Road Kabul 1001, Afghanistan; Kabul University of Medical Sciences Abu Ali Ibn Sina, University Road Kabul 1001, Kabul, Afghanistan

**Keywords:** neurofibroma, dumbbell neurofibroma, cervicothoracic spine, sporadic neurofibroma, massive neurofibroma, surgical extirpation, microsurgery

## Abstract

Sporadic cervicothoracic dumbbell neurofibromas are rare and surgically challenging when large. A 44-year-old male presented with left cervical swelling, cervicobrachial pain, and C8–T1 deficits with hypothenar atrophy. Magnetic resonance imaging revealed a 6.4 × 6.6 × 11 cm C4–D3 dumbbell mass without invasion. A single-stage posterior C6–D1 laminectomy with microsurgical excision and Cavitron Ultrasonic Surgical Aspirator–assisted debulking achieved complete resection. Postoperatively, pain resolved with marked neurological recovery. Histopathology confirmed WHO Grade I neurofibroma. This case shows that even giant lesions can be safely managed with meticulous microsurgical planning.

## Introduction

Neurofibromas are benign peripheral nerve sheath tumors, usually linked to NF1 but rare as solitary cervical lesions. Dumbbell tumors extend through neural foramina and can compress neural and vascular structures, causing pain and deficits [1, 2].

Management requires complete excision with functional preservation, though optimal strategy for giant cervicothoracic cases is unclear.

We report a 44-year-old male with a C4–D3 sporadic dumbbell neurofibroma treated via single-stage posterior microsurgical resection.

## Case report

### Clinical presentation

A 44-year-old male laborer presented with a 4-year history of a progressively enlarging painless left cervical mass as shown in Fig. 1. Over the past year, he developed cervicobrachial pain with C8–T1 sensory symptoms, progressive hand weakness, and hypothenar atrophy. No history of NF or genetic disorder; consent obtained.

**Figure 1:**
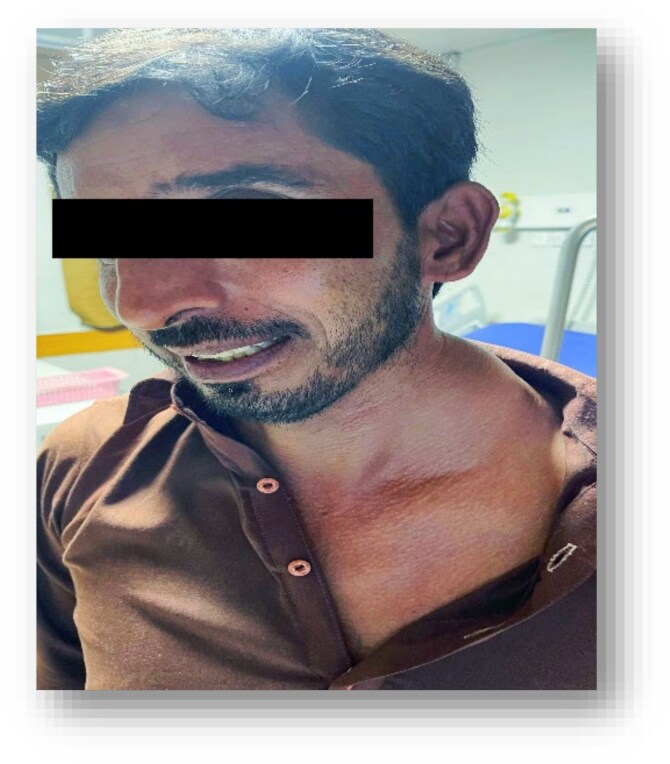
Clinical photograph demonstrating the conspicuous, solitary, ovoid neoplastic mass manifesting in the inferolateral aspect of the patient’s left cervical region, extending into the supraclavicular fossa and lower cervical triangle. The overlying integument appeared physiologically normal, devoid of cutaneous stigmata.

Examination showed a firm, non-tender 7 × 6.5 cm left supraclavicular mass deep to sternocleidomastoid and immobile. Neurologically, there was 4/5 intrinsic hand weakness with hypothenar atrophy and reduced sensation in the medial forearm and fifth digit; other findings were normal.

### Diagnostic investigations

Baseline labs were unremarkable. Magnetic resonance imaging (MRI) showed a well-circumscribed encapsulated C4–D3 mass (6.4 × 6.6 × 11 cm) with dumbbell configuration and C7–D1 foraminal extension, T1 hypointense, T2 hyperintense, and heterogeneously enhancing. The cord was displaced without compression or signal change, as shown in Figs 2–4.

**Figure 2:**
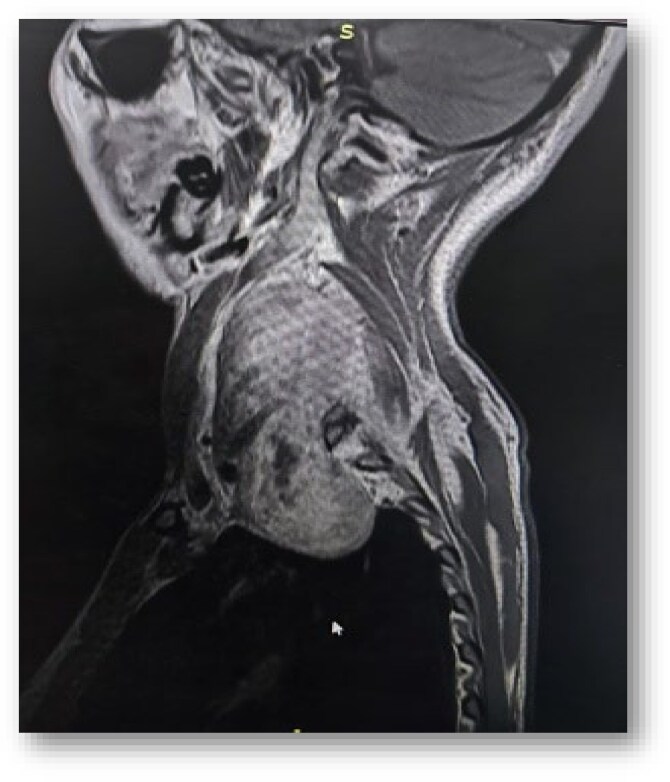
Sagittal T1-weighted MRI of the cervicothoracic spine demonstrating a large, elongated, predominantly hypointense mass extending from the C4 to D3.

**Figure 3:**
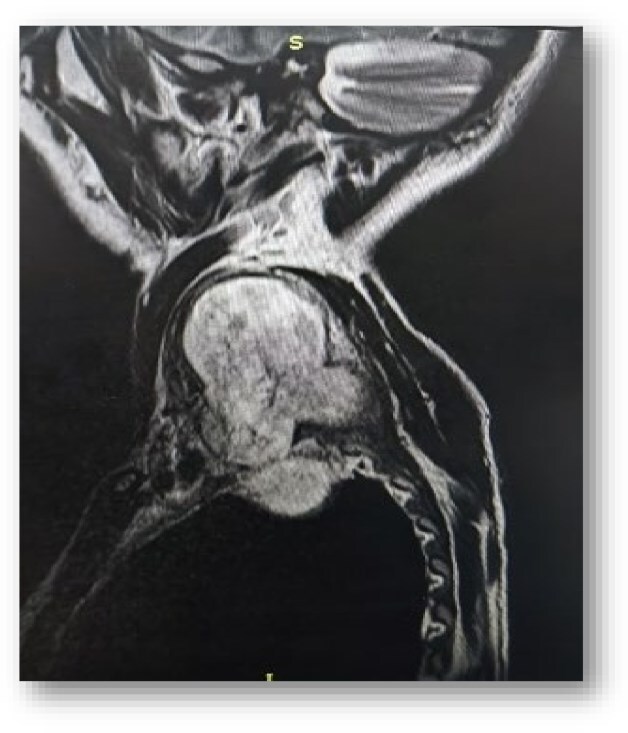
Sagittal T2-weighted MRI of the cervicothoracic spine revealing the extensive craniocaudal dimension of the markedly hyperintense neurofibroma, spanning C4 to D3.

**Figure 4:**
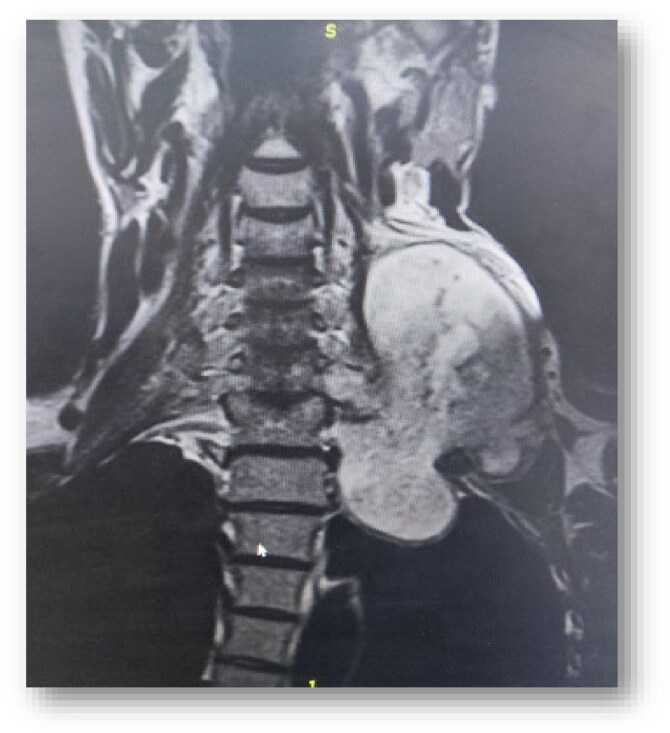
Coronal T1-weighted MRI with gadolinium contrast, illustrating the significant craniocaudal (C4–D3) and mediolateral extent of the heterogeneously enhancing neurofibroma; shows the typical “dumbbell” shape.

Computed tomography angiography (CTA) showed anteromedial displacement of the carotid artery and internal jugular vein with preserved fat planes and no invasion, confirming resectability. A preoperative diagnosis of a large sporadic neurofibroma was made.

### Surgical technique, postoperative course, and follow-up

The patient was positioned prone with Mayfield fixation, and intraoperative neuromonitoring with Somatosensory Evoked Potentials and Motor Evoked Potentials (SSEPs and MEPs) was used. A posterior midline approach from C5–D2 with C6–D1 laminectomies exposed the extradural tumor.

The lesion was a well-encapsulated gray-white mass, internally debulked using Cavitron Ultrasonic Surgical Aspirator (CUSA) to facilitate safe dissection. It arose from and involved the C8 and T1 roots; the C7 root was preserved. C8 and T1 were sacrificed for complete excision.

Dura was intact, with 400 ml blood loss and a 5-h operative time. Wound was closed over a drain.

Postoperatively, recovery was uneventful with mobilization on Day 1 and complete pain relief. Histopathology confirmed WHO Grade I neurofibroma with S-100 positivity.

At 12 months, the patient remained pain-free with improved hand strength (4 ± 5 MRC- Medical Research Council Scale), reduced sensory deficit, and full return to work with good functional outcome.

## Discussion

The successful resection of a giant, sporadic cervicothoracic dumbbell neurofibroma presents a significant neurosurgical challenge. This case is instructive due to the tumor’s large size, its complex anatomical location, and the excellent functional outcome achieved through a meticulously planned single-stage posterior approach.

### Epidemiology and pathophysiology

Spinal neurofibromas are benign nerve sheath tumors, commonly seen in NF1 but rare as solitary sporadic lesions. Giant cervical cases are exceptional. They consist of Schwann cells, perineurial-like cells, and fibroblasts in a collagenous/myxoid stroma, showing an infiltrative pattern with nerve fascicle involvement, unlike schwannomas, which are encapsulated and often nerve-sparing [3, 4].

### Clinical presentation and diagnostic imaging

A 44-year-old male presented with a 4-year history of a progressively enlarging painless left cervical mass. He developed cervicobrachial pain, C8–T1 sensory symptoms, hand weakness, and hypothenar atrophy. No Neurofibroma (NF) or genetic history; consent obtained.

Examination revealed a firm, non-tender 7 × 6.5 cm left supraclavicular mass. Neurologically, there was 4/5 intrinsic hand weakness with hypothenar atrophy and reduced sensation in the medial forearm and fifth digit.

### Surgical management of giant cervical dumbbell neurofibromas

Surgical resection is the treatment of choice for spinal neurofibromas, aiming for complete removal, decompression, and preservation of function and stability [5]. Approach selection depends on tumor location and extent [1]. A single-stage posterior approach was used here due to a predominantly extradural posterior–lateral lesion without ventral involvement or instability.

Key challenge is safe bulk reduction without injury. Microsurgery with CUSA-assisted internal decompression facilitated capsule relaxation and safer dissection from the dura and surrounding structures [6].

This “inside-out” technique minimizes spinal cord retraction and is a key principle in modern spinal tumor surgery [7]. Nerve root involvement is also critical, as neurofibromas often infiltrate their nerve of origin; thus, complete excision frequently requires sacrifice of the involved root, as supported in the literature [8, 9]. In this case, the C8 and T1 roots were sacrificed due to tumor involvement, while the displaced but uninvolved C7 root was preserved.

Post-laminectomy stability is an important consideration after multilevel decompression. Although kyphosis is a known risk, it is less frequent in non-dystrophic spines when facets and stabilizing structures are preserved [10, 11]. As there was no preoperative deformity and the facets were intact, no instrumentation was required [5].

## Conclusion

This case demonstrates successful management of a massive sporadic cervicothoracic dumbbell neurofibroma. With detailed MRI- and CTA-based planning and a single-stage posterior microsurgical approach with internal debulking, complete resection was achieved. The patient showed excellent neurological recovery, highlighting that even large complex tumors can be safely treated with functional preservation and low morbidity. This report adds to the limited evidence on surgical management of giant sporadic neurofibromas in non-NF1 patients.
